# Emergency Department Vital Sign Variability Is Associated with Hematoma Progression in Spontaneous Intracerebral Hemorrhage

**DOI:** 10.3390/jcm14134404

**Published:** 2025-06-20

**Authors:** Priya Patel, Abigail Kim, Milana Shapsay, Shriya Jaddu, Nahom Y. Seyoum, Anastasia Ternovskaia, Manahel Zahid, Hassan Syed, David Dreizin, Joshua Olexa, Afrah Ali, Stephanie Cardona, Quincy K. Tran, Jennifer A. Walker

**Affiliations:** 1 School of Medicine, University of Maryland, Baltimore, MD 21201, USA; pspatel@som.umaryland.edu (P.P.); nahoms1995@gmail.com (N.Y.S.); manahel.zahid@som.umaryland.edu (M.Z.); hassanksyed23@gmail.com (H.S.); 2 Research Associate Program in Emergency Medicine and Critical Care, Department of Emergency Medicine, University of Maryland School of Medicine, Baltimore, MD 21201, USA; kimay00@gmail.com (A.K.); mshapsay@gmail.com (M.S.); shriyajaddu@gmail.com (S.J.); aternovs@terpmail.umd.edu (A.T.); qtran@som.umaryland.edu (Q.K.T.); 3 Department of Radiology, University of Maryland School of Medicine, Baltimore, MD 21201, USA; ddreizin@umm.edu; 4 Department of Neurosurgery, University of Maryland School of Medicine, Baltimore, MD 21201, USA; jolexa@som.umaryland.edu; 5 Department of Emergency Medicine, University of Maryland School of Medicine, Baltimore, MD 21201, USA; aaali@som.umaryland.edu; 6 Department of Critical Care Medicine, Baptist Health System, Miami, FL 33176, USA; drscard013@gmail.com; 7 Program in Trauma, The R Adam Cowley Shock Trauma Center, University of Maryland School of Medicine, Baltimore, MD 21201, USA; 8 Department of Emergency Medicine, Baylor Scott & White All Saints Medical Center, Fort Worth, TX 76104, USA; 9 Department of Emergency Medicine, Burnett School of Medicine, Texas Christian University, Fort Worth, TX 76109, USA

**Keywords:** emergency department, critical care, intracranial hematoma, hemorrhagic stroke, hematoma progression, blood pressure variability, heart rate variability

## Abstract

**Background/Objectives**: Spontaneous intraparenchymal hemorrhage (sIPH) accounts for a significant proportion of strokes and is associated with an estimated 30-day mortality between 35 and 52%. Subsequent hematoma progression (HP) occurs in up to 30% of patients and is associated with blood pressure variability, increasing poor outcomes. This study evaluates systolic blood pressure and heart rate variability in the emergency department (ED) and HP in the first 24 h of admission. **Methods**: This retrospective study analyzed patients with sIPH presenting to the ED and transferred to a resuscitation unit between 2017 and 2020. Outcomes included the occurrence of HP. Variables included blood pressure variability as measured by the standard deviation in systolic blood pressure (SBP-SD), successive variation of systolic blood pressure (SBP-SV), standard deviation of heart rate (HR-SD), and successive variation of heart rate (HR-SV). Bivariate analysis and machine learning algorithms were used to identify ED predictors for HP. **Results**: Of the 142 records analyzed, 41 (29%) patients experienced HP. The medians [interquartile (IQR)] for baseline characteristics were similar between groups. In the group with no HP (control), the median [IQR] for SBP-SD was 17.6 [11–26] compared with 20.5 [13.9–26.1, *p* = 0.25]. The median [IQR] for standard deviation in SBP-SV was 18 [11.4–25.4] for the control group and 19.8 [15.2–27.3, *p* = 0.19] for the HP group. While bivariate analysis did not show statistical difference for SBP-SD, SBP-SV, HR-SD, or HR-SV, machine learning algorithms identified SBP-SD, HR-SD, and HR-SV as clinically impactful on HP with good accuracy (92.59% and 79.31%). **Conclusions**: This study suggests that there are factors in hyperacute hemodynamic management in the ED associated with HP among patients with sIPH.

## 1. Introduction

Spontaneous intraparenchymal hemorrhage (sIPH), non-traumatic bleeding in the brain parenchymal tissue, accounts for 10–15% of all strokes and is associated with a 30-day mortality rate estimated to be between 35 and 52% [1]. Initial hematoma volume is the strongest predictor of 30-day mortality and overall functional outcome in sIPH patients [2]. Continued bleeding can be seen in up to one-third of patients and leads to hematoma progression (HP) [3], which even at small amounts can increase the likelihood of mortality and poor functional outcome [4]. The association between high blood pressure (BP) and worsening HP in sIPH patients has been previously described [5,6]. Other studies have demonstrated that early intensive BP control resulted in attenuation of HP [7,8]. However, the role of other BP parameters such as blood pressure variability (BPV) in HP has not been adequately explored. Components of blood pressure variability can be defined as the average of absolute differences between consecutive blood pressure measurements (successive variations in systolic blood pressure [SBP-SV]) or variations in SBP during a period of time (standard deviation [SBP-SD]) [9]. Blood pressure variability within and beyond 24 h of disease onset has been shown to be an independent risk factor associated with outcomes among sIPH patients [10,11,12], but these studies did not investigate whether BPV during a hyperacute phase, such as the short period while patients are cared for in the emergency department, is associated with HP.

When patients with sIPH, particularly those with signs and symptoms of intracranial hypertension, present to an emergency department (ED) without neurosurgical capability, they are transferred to centers with neurosurgical capability for higher level of care. Previous studies suggest an association between ED BPV and subsequent development of acute kidney injury among patients with sIPH and subarachnoid hemorrhage (SAH) or between ED BPV and HP among patients with traumatic brain injury [4,11]. The association between ED BPV and the development of HP among sIPH patients, however, has not been described. We hypothesized that BPV during a short ED stay prior to transferring to a tertiary care center would be associated with HP after arrival to a neurosurgical center. In this study, we aimed to investigate the association between BPV and HP in patients with sIPH during this hyperacute phase. Furthermore, we explored the association between medical interventions and BPV during the ED course.

## 2. Materials and Methods

### 2.1. Study Setting and Patient Selection

This was a retrospective observational study from 1 January 2017 to 31 December 2020. Most data were retrospectively collected from a limited dataset that was prospectively maintained by the Department of Neurosurgery as part of their clinical care for patients with sIPH. All adult patients who were transferred to an academic quaternary center (UMMC) for the management of sIPH were eligible. This medical center has 24/7 neurosurgical coverage who are immediately available for necessary surgical interventions. When patients with sIPH present to our ED or are transferred from another ED without neurosurgery coverage, they are evaluated immediately by the on-call neurosurgery team. Further management will depend on the decision of the neurosurgery and critical care teams.

Patients undergo a second computer tomography (CT) scan of their brains between 6 and 24 h of admission to assess the stability of the hematoma. Thus, we included patients who had 2 consecutive CT scans within 24 h of admission. Patients who did not have sufficient ED records, those with traumatic hemorrhage, and those who developed hemorrhage secondary to malignancy, arteriovenous malformations, or ischemic stroke were excluded as the pathophysiology and outcomes of these patients are different from those with sIPH [9]. We also excluded patients who did not have intraparenchymal hemorrhage and patients with SAH.

The study protocol was reviewed and approved by the Principal Investigator’s Institutional Review Board (HP-00084554). No formal consent was required due to the observational nature of this study.

### 2.2. Outcome Measures

The primary outcome was rate of HP, which was defined as hematoma growth between the initial (baseline) CT scan at presentation and the follow-up CT scan, which is normally acquired per protocol within 24 h of admission. We defined hematoma growth on CT when the second volume was ≥30% compared with the baseline or when the change of hematoma volumes between the two CT scans exceeded 12.5 milliliters (ml) [13]. For hematoma volume, an attending radiologist reviewed the CT scans and calculated the hematoma volume according to the method ABC/2 [14]. The attending radiologist also determined whether the hemorrhage originated from an infratentorial location. The secondary outcome was the number of patients requiring external ventricular drain (EVD), which was documented by the neurosurgical team in the electronic medical record. Other outcomes included the degree of BPV as measured by the standard deviation in systolic blood pressure (SBP-SD) and successive variation of systolic blood pressure (SBP-SV) during the patient’s ED stay. Although a previous study has demonstrated that BPV during ED stay is associated with poor outcomes in ICH patients [15], the population in that study included patients with SAH and sIPH. It is essential to understand which components of ED care were associated with a greater degree of BPV as an important first step in addressing this issue. Other secondary outcomes included the predictors associated with either HP by percentage changes (≥30%) or and by absolute volume change (≥12.5 mL).

### 2.3. Data Collection and Management

Investigators who were not blind to the study hypothesis were trained in data extraction by the senior investigator, utilizing sets of 5 patient charts until at least 90% inter-rater agreement was achieved. Data were then extracted into a standardized Microsoft Excel spreadsheet (Microsoft Corp., Seattle, WA, USA). To reduce bias, investigators extracted data in independent sections. For example, investigators extracting interventions would not extract both blood pressure and outcomes data and vice versa. To increase dataset reliability, another investigator independently checked 20% of the data to maintain 90% inter-rater agreement during the data collection phase.

Sources of data were primarily from the electronic medical record at our institution, which uses Epic (Epic Systems Corp., Verona, WI, USA), and a neurosurgical limited dataset that was maintained prospectively as part of that team’s clinical care. This limited dataset included date and time of EVD insertion, EVD opening pressure, and whether there was intraventricular hemorrhage. When patients transferred from another ED not using Epic, accompanying paper records were utilized. Other ED clinical values that were collected included blood pressure measurements, presence of mechanical ventilation, and presence of seizures. We also collected components of the intracerebral hemorrhage (ICH) score (age, ICH volume, presence of intraventricular hemorrhage and infratentorial hemorrhage). Therapies in the ED including medications, blood products, and intravenous crystalloids were extracted. Medications that were ordered but not administered were not extracted.

### 2.4. Blood Pressure Variability (BPV)

**Figure A1:**
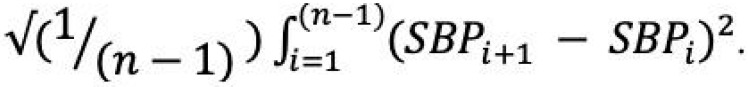
Calculation of successive variations in systolic blood pressure (SBP-SV).

**Figure A2:**
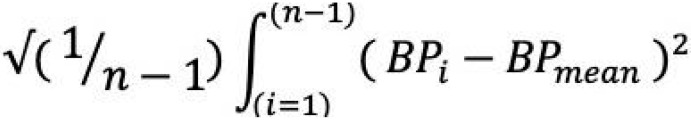
Calculation of standard deviation of systolic blood pressure (SBP-SD).

We collected all recorded blood pressure measurements while patients were in the ED starting from triage until the patients left the ED. We calculated BPV in the ED as previously described for successive variation in systolic blood pressure (SBP-SV) and standard deviation in systolic blood pressure (SBP-SD) [10,11,12,16]. The standard deviation in systolic blood pressure (SBP-SD) was defined as the average of the absolute differences from each blood pressure measurement to the mean of those measurements and effectively was a measure of how “tightly” clinicians controlled a patient’s SBP. The successive variation in systolic blood pressure (SBP-SV), which is effectively a measure of how “quickly” clinicians lower a patient’s SBP, was the average of absolute differences between consecutive blood pressure measurements. From all of the recorded blood pressure measurements, in addition to calculating SBP-SD and SBP-SV, we also identified the highest (SBP_max_) and lowest (SBP_min_) SBP values for each patient. Standard deviation of ED heart rate (HR-SD) and successive variation of ED heart rate (HR-SV) were calculated in a similar fashion. Calculations for SBP-SV and SBP-SD are demonstrated in Appendix A, Figure A1 and Figure A2, respectively.

### 2.5. Data Analysis

We did not perform sample size analyses due to the retrospective nature of this study. On the other hand, we used post hoc power analysis to assess our power to compare components of BPV between subgroups of patients with and without HP. G*Power version 3.1 [17] was used for post hoc power analysis.

Descriptive analyses were used to present data from our patients. Prior to analyses, histograms of continuous variables were inspected for their patterns of distributions. Normally, distributed data is presented as mean (±standard deviation [SD]) and was analyzed by the Student T test. Non-normally distributed data was expressed as the median (interquartile range [IQR]) and was analyzed by the Mann–Whitney U test. Categorical variables are presented with percentages and were compared by the Pearson’s chi-square tests. Comparisons between groups are expressed as the differences between groups and the 95% confidence intervals (95% CI) of the differences.

### 2.6. Machine Learning Algorithms

For predictors associated with the development of HP and EVD placements in our patient population, we used both the random forest (RF) and XGBoost machine learning algorithms. We used RF as our primary method to identify the predictors associated with our outcome. We subsequently used XGBoost in order to validate our results by RF. The purpose of this approach was to confirm that our results were valid and that there were no differences in statistical methodology [18]. Thus, predictors that were identified by both models would be considered as significant predictors for the outcome of interest. Both RF and XGBoost models were constructed using Scikit-Learn Python libraries, version 3.9.12: one model was constructed per outcome. The model dataset included a total of 25 variables (Appendix B). A random selection of 80% of the data (105 patients) formed the training dataset, and the other 20% of the data (26 patients) comprised the test dataset. Each of the models was trained using tenfold cross-validation to mitigate overfitting, an issue that can arise with smaller datasets. In tenfold cross-validation, the training dataset is split into ten subsets, and the model is trained using nine of the subsets and validated using the tenth subset. This process of training and validation is repeated ten times, and the model’s performance on all of the folds is utilized to tune the model’s hyperparameters. The model was then evaluated on the test set, and accuracy and F1 scores were calculated. Accuracy is a high-level measurement of model performance, and the F1 score is an effective measure of model performance for uneven class distributions. Both F1 score and accuracy have a maximum possible value of 1 (perfect prediction) and a minimum possible value of 0 (no correct prediction). Additionally, Brier scores were calculated in order to assess model calibration and refinement. A Brier score of 0 is considered perfect accuracy and calibration, whereas a Brier score of 1 is imperfect accuracy and calibration.

After the models were assessed based on accuracy and F1 scores and tuned as needed, the Shapley additive explanations (SHAP) feature selection function was employed. SHAP uses a game theory approach to measure each variable’s contribution to the final outcome and represents each of these contributions as a SHAP value. For each model, the mean of the absolute SHAP values (one SHAP value was generated per prediction) was calculated, and the top five predictors were identified. 

For sensitivity analysis for each outcome, an additional model was created, trained on only its top five predictors, and evaluated; the accuracies of these models were then compared with the accuracies of the original models (all variables included), thus confirming whether the top five predictors were indeed significant predictors for the outcome of interest.

Statistical analyses were performed with Minitab version 20 (Minitab, LLC, State College, PA, USA). RF and XGBoost modeling was performed with Python version 3.9.12 (www.python.org) in Spyder IDE (www.spyder-ide.org). Data preprocessing was performed using Pandas and NumPy, models were created using Scikit-Learn, and all feature analysis was completed using Shapley additive explanations. All statistical analyses with *p*-value < 0.05 were considered significant.

## 3. Results

### 3.1. Patient Characteristics 

**Figure 1:**
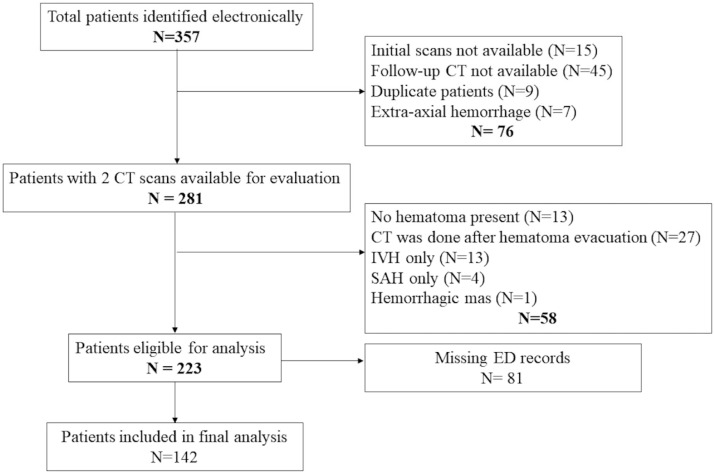
Patient flow diagram.

Three hundred fifty-seven (357) patients were initially identified, and 142 patients were included in the final analysis (Figure 1). Forty-one (29%) patients were noted to have hematoma progression (+HP), whereas 101 patients (71%) did not have hematoma progression (-HP). In comparison, both groups had similar baseline characteristics (Table 1). There were approximately 19% more patients with +HP who required invasive mechanical ventilation in the ED, and this difference was statistically significant (95% CI −0.37 to −0.01, *p* = 0.04) (Table 1). In the bivariate analysis, there was no statistically significantly difference for SBP-SV between both groups (difference between groups −2.12, 95% CI −5.77 to 1.54, *p* = 0.25) (Table 2), nor for SBP-SD, HR-SV, and HR-SD.

The post hoc power analysis for comparison of the means of SBP-SV and SBP-SD between both groups of patients suggested that our study would have a power of 95%, at an alpha of 0.05 to detect the current statistical differences, if there was a statistical significance in means between SBP-SV and SBP-SD among both groups. On the other hand, bivariate analysis suggested that patients with +HP had significantly higher mortality (difference between groups 17%, 95% CI −0.34 to −0.01, *p* = 0.04) (Table 2).

### 3.2. Primary Outcome: Rate of Composite Hematoma Progression by Either Volume or Absolute Percentage Changes

The top features impacting the HP model included SBP-SV, ED serum sodium, SBP-SD, HR-SD, and HR-SV (Table 3A). The RF algorithm analysis demonstrated very good accuracy (92.59%) with an F1 score of 0.9202. The XGBoost algorithm analysis demonstrated that the top features that had an impact on the HP model were ED serum creatinine, ED serum sodium, HR-SD, SBP-SD, and HR-SV (Table 3B). This XGBoost model demonstrated good accuracy and F1 score (79.31%, 0.78, respectively).

**Figure 2:**
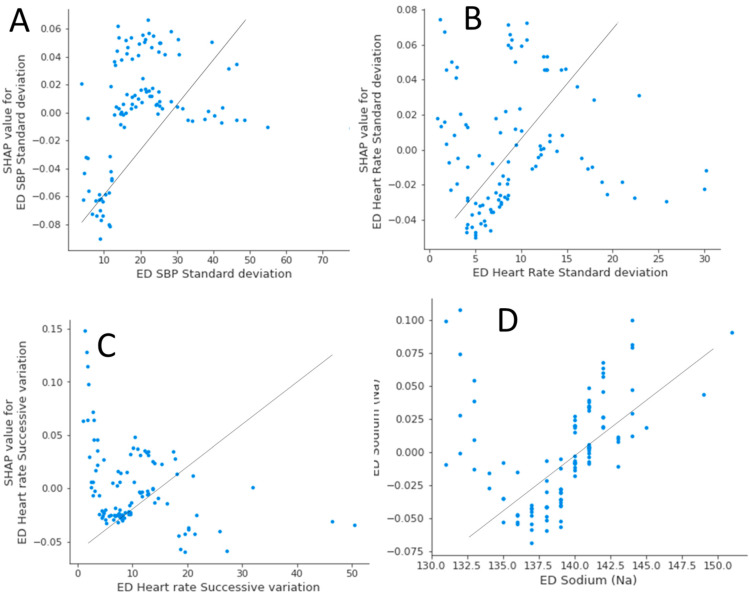
Correlation graph demonstrating Shapley additive explanations (SHAP) features (*Y*-axis) and values of significant predictors (*X*-axis), being identified by both machine learning algorithms, for hematoma progression. (**A**) Higher SBP-SD values correlated with higher positive SHAP values and higher likelihood of developing hematoma progression. (**B**) Higher HR-SD values correlated with higher positive SHAP values and higher likelihood of developing hematoma progression. (**C**) Higher HR-SV values correlated with higher positive SHAP values and higher likelihood of developing hematoma progression. (**D**) Higher serum sodium values correlated with higher positive SHAP values and higher likelihood of developing hematoma progression.

Thus, both models agreed that SBP-SD, HR-SD, HR-SV, and ED serum sodium were the highest risk factors for HP. The correlation graphs suggest that higher SBP-SD, HR-SD, HR-SV, and higher ED serum sodium predict a higher likelihood of having HP (Figure 2A–D). Because ED serum creatinine was not identified by the RF algorithm for composite HP by either volume expansion or absolute percentage changes, it was not considered predictive of HP.

### 3.3. Secondary Outcome: The Need for EVD Placement

There were 45 (32%) patients who required EVD placement upon arrival to the study center. The top features impacting EVD placement, which were identified by the RF model, included ICH score, ED serum sodium, need for invasive mechanical ventilation in the ED, SBP-SV, and SBP-SD (Table 3A). The RF algorithm analysis demonstrated a very good accuracy of 81.48% and a very good F1 score of 0.8034.

The XGBoost algorithm analysis demonstrated the top risk factors for EVD placement, including ICH score, SBP-SD, ED serum sodium level, HR-SD, and ED serum glucose. This XGBoost model was associated with a good accuracy of 82.76% and a good F1 score of 0.8329 (Table 3B). 

**Figure A3:**
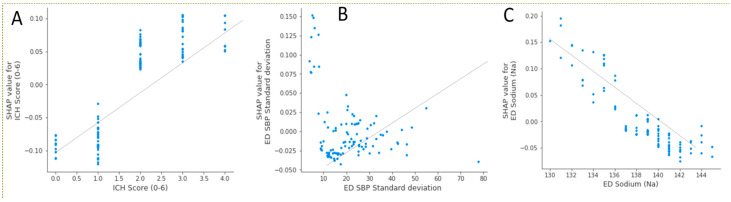
Correlation graph demonstrating Shapley additive explanations (SHAP) features (*Y*-axis) and values of significant predictors (*X*-axis), as identified by both machine learning algorithms, for the need of external ventricular drain. (**A**) Higher intracerebral hemorrhage score (ICH score) correlated with higher positive SHAP values and higher likelihood of requiring EVD. (**B**) Higher SBP-SD values correlated with higher positive SHAP values and higher likelihood of requiring EVD. (**C**) Higher serum sodium values correlated with more negative SHAP values and lower likelihood of requiring EVD.

Both algorithms agreed that ICH score, ED serum sodium, and SBP-SD were the top predictors for EVD placement. The correlation graph (Appendix C, Figure A3A–C) suggested that higher ICH score and SBP-SD would predict increased risk for EVD. In contrast, higher sodium levels would be associated with negative SHAP values and predict a lower likelihood of requiring EVD (Appendix C, Figure A3C).

### 3.4. Secondary Outcome: Hematoma Progression as Defined by Absolute Volume Change (≥12.5 mL)

For this secondary outcome, the RF algorithm analysis suggested that the top features that had an impact include higher sodium levels, ED requirement for invasive mechanical ventilation, SBP-SV, total number of heart rate measurements during ED stay, and serum glucose (Table 3). The RF algorithm demonstrated a very good accuracy of 96.30% and a very good F1 score of 0.9572. 

The XGBoost model identified the top features that had an impact on the absolute hematoma volume change, which include: the total number of heart rate measurements during ED stay, HR-SV, ED requirement for invasive mechanical ventilation, the total number of blood pressure measurements during ED stay, and higher serum sodium (Table 3B). The XGBoost algorithm analysis demonstrated a very good accuracy of 93.10% with a very good F1 score of 0.8978. Both algorithms identified higher sodium levels, invasive mechanical ventilation, and the total number of heart rate measurements during the ED stay were among the top risk factors for this outcome.

### 3.5. Secondary Outcome Hematoma Progression by Percentage Change (≥30%)

RF algorithms indicated that the top features that had an impact on HP by percentage change include SBP-SV, serum glucose, serum sodium, HR-SD, and serum creatinine, all of whose higher values will predict a higher probability of HP by volume change (≥30%). This RF algorithm analysis demonstrated a very good accuracy of 85.19% and a good F1 score of 0.7937 (Table 3A). The top features that had an impact on HP by percentage change include serum creatinine, serum sodium, SBP-SD, HR-SV, and serum glucose. The XGBoost algorithm analysis demonstrated a good accuracy of 79.31% with a good F1 score of 0.7497 (Table 3B). Therefore, both algorithms identified serum glucose, sodium, and creatinine as among the top features that would have predictors of patients who will have developed hematoma volume change.

## 4. Discussion

In this retrospective, single-center study, the association of ED blood pressure variability (BPV) on hematoma progression (HP) was examined. Our multi-approach analysis identified several variables and components of BPV in the ED that could predict HP or the need for EVD placement within 24 h of patients presenting to the ED.

Our bivariate analysis did not show statistical significance in blood ED BPV and outcomes. Based on our post hoc power analysis, however, it could be possible that there was a very small difference between SBP-SV or SBP-SD and patients with +HP when compared with those patients with -HP. Nonetheless, our RF algorithm, after considering many other clinical confounders, demonstrated that SBP-SD is an important predictor for HP. While our results agreed with previous findings that BPV would be associated with poor outcomes for patients with sIPH [10,11], our findings are important because this study collected blood pressure measurements during ED stay rather than an entire 24 h period as in previous studies. This suggests that hyperacute management of blood pressure variables at ED arrival is important.

The results from this study suggest that fluctuations of blood pressure during a short period of time during the hyperacute phase in the ED strongly predict HP. Thus, until further studies confirm or refute these findings, ED clinicians may consider early anti-hypertensive infusions to steadily control blood pressure among patients with sIPH. In another study, starting nicardipine early was also associated with lower odds of acute kidney injury among patients with spontaneous intracranial hemorrhage [19], suggesting other benefits of anti-hypertensive infusions. 

The pathophysiology of BPV in patients with sICH is unclear. First, BPV could be iatrogenic, as previous studies suggest that patients not receiving adequate analgesia and sedation experience higher BPV [16,20]. This may suggest why our study found that patients requiring invasive mechanical ventilation in the ED were associated with +HP. Factors such as pain and inadequate analgesia can result in hypertension or blood pressure variability. One small study found an association between vital sign changes and acute pain in acute brain injury patients [21]. Another small retrospective study looked at factors that can impact BPV in patients with spontaneous ICH and found that adequate sedation, hyperosmolar therapy, and fluid resuscitation were associated with lower BPV [20]. Not only does treating pain and providing sedation potentially reduce BPV, analgesia and appropriate sedation use is a strong recommendation in critically ill patients, and protocolized treatment can improve outcomes [22]. This should be taken into consideration in the hyperacute phase of patients with ICH and would influence pre-hospital and ED treatment protocols of these patients.

Autonomic dysregulation could be another cause for BPV and subsequent HP. A previous study suggested that even subclinical brain damage that was only detected by magnetic resonance imaging was associated with high BPV [23]. Thus, BPV was hypothesized to be a manifestation of autonomic dysregulation [24,25], which in turn exacerbated bleeding. Our study suggests that both BPV and HRV are predictors for HP, supporting the hypothesis that HP is associated with autonomic dysregulation, manifesting as BPV and HRV. There appears to be an association between BPV and hematoma progression, as BPV might increase oncotic and hydrostatic pressure gradients in the perihematomal region, which would exacerbate cerebral edema [26]. However, a reverse association where hematoma progression could occur before BPV is also possible as hematoma progression may impair cerebral autoregulation [26]. For example, because the thalamus regulates blood pressure [27], a hemorrhagic thalamus could be associated with high BPV.

### Limitations 

There are several limitations to this study. One limitation is that it is a single-center study occurring in a large, quaternary care center. These results, therefore, may not be generalizable to other populations or centers. There was also a high rate of exclusions in our patient sample, which could contribute to lower generalizability compared with all ICH patients. Further research in this field with a larger, more diverse sample is imperative as this can produce more generalizable results. ICH is a pathology that accounts for a large proportion of strokes and has a high mortality rate; therefore, it is important to create a better understanding of factors that impact outcomes and patient care.

The blood pressure data was heterogeneous due to the dynamic processes of blood pressure monitoring in critically ill patients, such as in patients with sIPH. For example, patients transported to radiology for imaging may have other factors impacting blood pressure measurement intervals. If there is a longer interval between measurements or if the measurements have not been documented appropriately, there may be an impact on what is documented in the electronic medical record. True BPV may be missed due to this systematic issue, although we did use the total number of blood pressure measurements as a surrogate marker for a patient’s dynamic blood pressure management in the ED.

Furthermore, we could not take into account the variability and management during transport between outside hospitals and the study center, as documentation from transport teams can be limited. Additionally, most patients were transferred to our facility from other hospitals, and therefore, there could be differences in the management and documentation of care prior to arrival to our institution. The ability to determine whether observed hemodynamic variables are causative factors in HP or whether this is an association only is a limitation due to the retrospective nature of our study. Furthermore, our study is vulnerable to selection bias due to unblinded researchers; however, measures as described were instituted to limit bias.

## 5. Conclusions

This study suggests that components of blood pressure variability and heart rate variability in the emergency department are predictors for hematoma progression among patients with spontaneous intraparenchymal hemorrhage. Further studies are necessary to evaluate this concept on a larger scale with a larger patient population and to examine other factors in the emergency department that could have had an impact on HP in patients with intraparenchymal hemorrhages.

## Figures and Tables

**Table 1:** Demographics and interventions for patients presenting to ED with spontaneous intraparenchymal hemorrhage.

Parameters	All Patients	No HP	Yes HP	Difference Between Group	95% CI	*p*
	N = 142	N = 101	N = 41			
**Demographics**						
Age, years, mean (SD)	62.6 (14.8)	62.1 (15.6)	64 (12.6)	−1.84	(−6.81, 3.13)	0.46
Age (≥80 years), N (%)	18 (12.7)	13 (12.9)	5 (12.2)	0.01	(−0.11, 0.13)	0.91
**Past medical history, N (%)**						
CAD	15 (10.6)	12 (11.9)	3 (7.3)	0.05	(−0.06, 0.15)	0.55
DM	37 (26.1)	30 (29.7)	7 (17.1)	0.13	(−0.02, 0.27)	0.09
HTN	102 (71.8)	71 (70.3)	31 (75.6)	−0.05	(−0.21, 0.11)	0.51
CKD	13 (9.2)	9 (8.9)	4 (9.8)	−0.01	(−0.11, 0.10)	0.99
Any liver disease	2 (1.4)	1 (1)	1 (2.4)	−0.01	(−0.07, 0.04)	0.5
**Past medications, N (%)**						
Any antiplatelet	39 (27.5)	28 (27.7)	11 (26.8)	0.01	(−0.15, 0.17)	0.91
Any anticoagulation	22 (15.5)	16 (15.8)	6 (14.6)	0.01	(−0.12, 0.14)	0.86
**Clinical characteristics on ED admission**						
Sodium (mEq/L), mean (SD)	139.1 (3.6)	139.2 (3.3)	139.1 (4.3)	0.13	(−1.36, 1.62)	0.86
Creatinine (mg/dL), median [IQR]	1 [0.8–1.2]	1 [0.8–1.2]	1 [0.8–1.3]	−0.06	(−0.20, 0.08)	0.4
Glucose (mg/dL), median [IQR]	139.5 [118–177.3]	140 [120–182]	137 [111.5–162.5]	12	(−3, 27)	0.13
**Other clinical characteristics**						
GCS on ED triage, median [IQR]	13 [8.8–15]	13 [9–15]	12 [6.5–15]	1	(0, 2)	0.09
ICH volume ≥ 30 mL, N (%)	50 (35.2)	37 (36.6)	13 (31.7)	0.05	(−0.12, 0.22)	0.57
Intraventricular hemorrhage, N (%)	83 (58.5)	62 (61.4)	21 (51.2)	0.1	(−0.08, 0.28)	0.27
Infratentorial bleed, N (%)	27 (19)	18 (17.8)	9 (22)	−0.04	(−0.19, 0.11)	0.58
ICH score, mean (SD)	1.8 (1.1)	1.8 (1.1)	1.9 (1.2)	−0.11	(−0.53, 0.31)	0.6
Clinical seizure prior or during ED stay, N (%)	15 (10.6)	8 (7.9)	7 (17.1)	−0.09	(−0.22, 0.04)	0.16
Invasive mechanical ventilation during ED, N (%)	57 (40.1)	35 (34.7)	22 (53.7)	−0.19	(−0.37, −0.01)	0.04
Interval of triage to ED mechanical ventilation (minutes), median [IQR]	63 [36.5–116.5]	63 [45–111]	70 [30.8–118.3]	0	(−30, 29)	0.97
ED Length of stay (minutes), median [IQR]	174.5 [129.8–269.8]	181 [135–268.5]	150 [107.5–307.5]	15	(−27, 50)	0.44

Abbreviations: CAD = coronary artery disease, CKD = chronic kidney disease, DM = diabetes mellitus, GCS = Glasgow Coma Scale, HTN = hypertension, ICH = intracranial hemorrhage.

**Table 2:** Interventions and patient outcomes.

Medical Therapy						
Any crystalloids, N (%)	39 (27.5)	29 (28.7)	10 (24.4)	0.04	(−0.12, 0.20)	0.59
Any nicardipine, N (%)	60 (42.3)	47 (46.5)	13 (31.7)	0.15	(−0.02, 0.32)	0.09
Interval of triage to ED nicardipine infusion (minutes), median [IQR]	68 [33–133.5]	64 [29–130]	88 [47.8–146]	−18	(−58, 19)	0.31
Any clevidipine, N (%)	17 (12)	8 (7.9)	9 (22.5)	−0.15	(−0.29, −0.01)	0.04
Interval of triage to ED clevidipine infusion (minutes), median [IQR]	45 [27.5–76.5]	50 [33.8–110.5]	32 [13.5–67]	21	(−18, 69)	0.23
Both nicardipine and clevidipine, N (%)	1 (0.7)	1 (1)	0 (0)	0.01	(−0.01, 0.03)	0.99
**Any IV push, N (%)**	29 (20.4)	22 (21.8)	7 (17.1)	0.05	(−0.09, 0.19)	0.51
>1 IV push, N (%)	4 (2.8)	3 (3)	1 (2.4)	0.005	(−0.05, 0.06)	0.99
Interval of triage to first ED IV push (minutes), median [IQR]	66 [33–109]	62 [31–118.5]	68 [41–90]	−1	(−42, 59)	0.99
**Any seizure medication, N (%)**	99 (69.7)	69 (68.3)	30 (73.2)	−0.05	(−0.21, 0.11)	0.56
Any phenytoin, N (%)	4 (2.8)	2 (2)	2 (4.9)	−0.03	(−0.10, 0.04)	0.58
Any levetiracetam/Keppra, N (%)	96 (67.6)	68 (67.3)	28 (68.3)	−0.01	(−0.18, 0.16)	0.91
>1 seizure medication, N (%)	1 (0.7)	1 (1)	0 (0)	0.01	(−0.01, 0.03)	0.99
**Any hyperosmolar therapy, N (%)**	21 (14.8)	15 (14.9)	6 (14.6)	0.002	(−0.13, 0.13)	0.97
3% saline, N (%)	2 (1.4)	1 (1)	1 (2.4)	−0.01	(−0.07, 0.04)	0.5
Mannitol, N (%)	19 (13.4)	14 (13.9)	5 (12.2)	0.02	(−0.10, 0.14)	0.79
**Any blood product, N (%)**	12 (8.5)	7 (6.9)	5 (12.2)	−0.05	(−0.16, 0.06)	0.36
Fresh frozen plasma, N (%)	2 (1.4)	1 (1)	1 (2.4)	−0.01	(−0.07, 0.04)	0.5
Platelets, N (%)	3 (2.1)	1 (1)	2 (4.9)	−0.04	(−0.11, 0.03)	0.2
PCC, N (%)	9 (6.3)	7 (6.9)	2 (4.9)	0.02	(−0.06, 0.10)	0.99
**Blood pressure variability**						
SBP standard deviation, median [IQR]	18.7 [12.1–26]	17.6 [11.5–26]	20.5 [13.9–26.1]	−2.12	(−5.77, 1.54)	0.25
SBP successive variation, median [IQR]	18.4 [12.5–25.9]	18 [11.4–25.4]	19.8 [15.2–27.3]	−2.55	(−5.77, 1.06)	0.19
**Heart rate variability**	N = 132	N = 92	N = 40			
Heart rate standard deviation, median [IQR]	7.9 [4.7–12.2]	7.7 [4.8–11.8]	8.9 [4–12.9]	−1	(−3.23, 1.08)	0.31
Heart rate successive variation, median [IQR]	8.5 [4.6–12.9]	8.1 [4.7–12.7]	10.3 [3.8–13.5]	−0.43	(−3.01, 1.75)	0.74
**Interventions at quaternary care center**						
Any neurosurgical interventions, N (%)	56 (39.4)	43 (42.6)	13 (31.7)	0.11	(−0.06, 0.28)	0.22
Any EVD, N (%)	45 (31.7)	34 (33.7)	11 (26.8)	0.07	(−0.10, 0.23)	0.41
Any craniectomy, N (%)	9 (6.3)	6 (5.9)	3 (7.3)	−0.01	(−0.11, 0.08)	0.72
Any craniotomy, N (%)	26 (18.3)	18 (17.8)	8 (19.5)	−0.02	(−0.16, 0.13)	0.82
ICP * (cm H20), median [IQR]	24 [15–30]	20 [13.8–28]	30 [15–30]	−5	(−12, 0)	0.05
**Hospital disposition, N (%)**						
Discharge home	21 (14.8)	12 (11.9)	9 (22)	−0.1	(−0.24, 0.04)	0.16
Discharge to any rehabilitation facility	72 (50.7)	55 (54.5)	17 (41.5)	0.13	(−0.05, 0.31)	0.16
Discharge to skilled nursing facilities	18 (12.7)	17 (16.8)	1 (2.4)	0.14	(0.06, 0.23)	0.02
Hospice/Death	31 (21.8)	17 (16.8)	14 (34.1)	−0.17	(−0.34, −0.01)	0.04

* Only available from 45 patients who required EVD. Abbreviations: EVD = extraventricular drain, ICP = intracranial pressure, IV = intravenous, PCC = prothrombin complex concentrate, RBCs = red blood cells, SBP = systolic blood pressure.

**Table 3:** (**A**) Results from random forest (RF) models for each outcome of interest. Only the top 5 independent variables were reported. The Brier scores for the random forest models trained to predict the four outcomes are as follows: 0.1579, 0.1525, 0.1450, and 0.0865. Abbreviations: EVD = extraventricular drain, ICH = intracranial hemorrhage, SBP = systolic blood pressure. (**B**) Results from XGBoost models for each outcome of interest. Only the top 5 independent variables were reported. The Brier scores for the XGBoost models trained to predict the four outcomes are as follows: 0.2287, 0.1591, 0.1499, and 0.1000. Abbreviations: EVD = extraventricular drain, ICH = intracranial hemorrhage, SBP = systolic blood pressure.

A			
Outcome	Top Five Predictors	Mean (|SHAP Value|)	Model Performance
Hematoma progression (either absolute hematoma volume change or percentage change)	ED SBP Successive Variation	0.10	Accuracy: 92.59% F1 Score: 0.9202
	2. ED Sodium (Na)	0.07	
	3. ED SBP Standard Deviation	0.06	
	4. ED Heart Rate Standard Deviation	0.06	
	5. ED Heart Rate Successive Variation	0.06	
The need for EVD placement	ICH Score	0.14	Accuracy: 81.48% F1 Score: 0.8034
	2. ED Sodium	0.10	
	3. ED Mechanical Ventilation	0.09	
	4. ED SBP Successive Variation	0.07	
	5. ED SBP Standard Deviation	0.06	
Hematoma progression by absolute hematoma volume change (≥12.5 mL)	ED Sodium (Na)	0.07	Accuracy: 96.30% F1 Score: 0.9572
	2. ED Mechanical Ventilation	0.06	
	3. ED SBP Successive Variation	0.05	
	4. Total Number of Heart Rate Measurements During ED Stay	0.05	
	5. ED Glucose (Glu)	0.05	
Hematoma progression by percentage change (≥30%)	ED SBP Successive Variation	0.10	Accuracy: 85.19% F1 Score: 0.7937
	2. ED Glucose	0.08	
	3. ED Sodium	0.07	
	4. ED Heart Rate Standard Deviation	0.07	
	5. ED Creatinine	0.07	
**B**			
**Outcome**	**Top Five Predictors**	**Mean (|SHAP Value|)**	**Model Performance**
Hematoma progression (either absolute hematoma volume change or percentage change)	ED Creatinine (Cr)	0.78	Accuracy: 79.31% F1 Score: 0.7804
	2. ED Sodium (Na)	0.71	
	3. ED Heart Rate Standard Deviation	0.71	
	4. ED SBP Standard Deviation	0.56	
	5. ED Heart Rate Successive Variation	0.49	
The need for EVD placement	ICH Score	1.23	Accuracy: 82.76% F1 Score: 0.8329
	2. ED SBP Standard Deviation	0.78	
	3. ED Sodium	0.76	
	4. ED Heart Rate Standard Deviation	0.50	
	5. ED Glucose	0.47	
Hematoma progression by absolute hematoma volume change (≥12.5 mL)	Total Number of Heart Rate Measurements During ED Stay	0.95	Accuracy: 93.10% F1 Score: 0.8978
	2. ED Heart Rate Successive Variation	0.74	
	3. ED Mechanical Ventilation	0.72	
	4. Total Number of Blood Pressure Measurements During ED Stay	0.56	
	5. ED Sodium (Na)	0.50	
Hematoma progression by percentage change (≥30%)	ED Creatinine (Cr)	0.67	Accuracy: 79.31% F1 Score: 0.7497
	2. ED Sodium (Na)	0.58	
	3. ED SBP Standard Deviation	0.49	
	4. ED Heart Rate Successive Variation	0.48	
	5. ED Glucose (Glu)	0.46	

## Data Availability

No new data was created or analyzed in this study.

## Abbreviations

The following abbreviations are used in this manuscript:

MDPI	Multidisciplinary Digital Publishing Institute
DOAJ	Directory of Open Access Journals

## Appendix B

**Table A1:** List of variables used for machine learning analysis (not in any particular order).

**Variables for Random Forest Modeling**
History of CAD
History of DM
History of HTN
History of CKD
History liver disease
Antiplatelet
Anticoagulation
Serum sodium (Na)
Serum creatinine (Cr)
Serum glucose (Glu)
Intracerebral hemorrhage score (ICH)
Seizure prior or during ED stay
Invasive mechanical ventilation
Any crystalloids
Nicardipine infusion
Clevidipine infusion
Both nicardipine and clevidipine
Any intravenous push
Any seizure medication
Any hyperosmolar therapy
Any blood product
Total number of ED systolic blood pressure (SBP) measurements
ED SBP standard deviation
ED SBP successive variation
Total number of ED heart rate measurements
ED heart rate standard deviation
ED heart rate successive variation
